# When I say … pragmatism

**DOI:** 10.1111/medu.70204

**Published:** 2026-03-11

**Authors:** Ghaith Alfakhry, Danica Sims, Ariel Lindorff

**Affiliations:** ^1^ Department of Education University of Oxford Oxford UK; ^2^ Association for Medical Education in Syria Damascus Syria; ^3^ Biomedical Engineering and Healthcare Technology Research Centre, Faculty of Health Sciences University of Johannesburg Johannesburg South Africa

## Abstract

Pragmatism isn't just a method—it's a philosophy that puts the research question first, bridges ontological divides, and empowers medical education scholars to transcend rigid paradigms. #MedEd

While designing a research project (G.A.) on self‐assessment of communication skills, I found myself navigating a deep ideological divide between two scholarly camps—each with starkly different views on reality (ontology), systems of knowledge (epistemology) and how research should be conducted (methodology).

The postpositivist camp believed in a *single* observer‐independent reality *measured objectively* using reliable *scientific* methods that put numbers (quantitative methods) on a pedestal. This all seemed too Pythagorean to me. Believing that an observer's reality can be captured entirely by a number struck me as a reductionist view of our complex value‐laden social reality. On the other hand, the interpretivist camp sounded too metaphysical to me. Interpretivists believed in *multiple* socially co‐constructed realities, s*ubjective* to the observer, who reflexively interprets the world using language, discourse and dialogue (qualitative methods).

These two camps often operated independently and justified their approaches in stark comparison with the other's worldview and research practices. Choosing one or the other of these paradigms when designing my research felt artificial and unnecessarily limiting. Quantitative research offered empirical validity, statistical generalisability and hypothesis testing. These are features valued by decision‐makers and suited to large samples. In comparison, qualitative research provided rich, contextual insights, captured participants' lived experiences and was flexible to changing study settings.

I struggled with this ‘either/or’ decision, as I was drawn to the strengths of both approaches and frustrated by the pressure to align philosophically and methodologically with one paradigm. Fortunately, there was a third camp which sought to integrate *both* qualitative and quantitative methodological and philosophical perspectives. It did not make itself as pronounced as the previous two. But when the qualitative/quantitative dualism was put under scrutiny, one could discern those scholars in the third camp. A particularly popular philosophical stance taken up by those in that third camp was *pragmatism*.

This piece provides arguments for *methodological* pragmatism in the practice of medical education research, to transcend the qualitative/quantitative *dualism* while also giving the reader some context for some of the existing philosophical debates and stances surrounding it.

Pragmatists highlight purists' ‘false dualism’ as described by Pring.^1^ Instead, they tend to be *methodological pluralists*. In so doing, the pragmatists reject the incommensurability thesis that argues that different paradigms and their associated methodologies/methods cannot be reconciled and cannot and should not be mixed in a single research project. Pragmatists integrate different methodologies and epistemologies in the sense that they relate dynamically to, build on, intersect or interact with one another but not in the sense that they converge on some single unified *truth*.

Methodological pragmatism's key tenet is ‘the dictatorship of the research question’.^2^ This maxim provides a practical rationale for bridging rigid paradigmatic divides, allowing the research question to dictate the research approach. Many pragmatists intentionally avoid engaging in ‘paradigm wars’, arguing that they do not have to justify their methodology based on broader philosophical assumptions—an approach Patton called a ‘paradigm of choices’.^3^ They challenge the assumption that epistemology necessarily determines methodology. Although pragmatism does have its philosophical foundation, researchers do not have to pledge allegiance to it to be allowed to use their methodological pluralism. This was made clear by Maxcy's distinction between *epistemological* and *methodological pragmatism*. While the former is related to pragmatists' theory of knowledge, the latter is more concerned with the pragmatists' logic and reasoning behind the selection of methods.^4^

Pragmatism as an all‐encompassing paradigm is a North American philosophy whose primary founders were Charles Sanders Peirce, William James and John Dewey who wrote extensively on it in the late 19th and early 20th century. Pragmatism, in simple terms, is a pluralistic worldview that avoids rigid philosophical dualisms and emphasises practical problem‐solving in real‐world contexts. It recognises *both* singular and multiple realities, combining objective *and* subjective elements, deductive *and* inductive reasoning, context‐specific *and* generalisable findings in a layered, experiential world. Pragmatism encompasses a variety of approaches to science, such as *instrumentalism*, which views scientific theories as useful *tools* rather than descriptions of the nature of reality. *Constructive empiricism* is another pragmatic philosophy of science which contends that mind‐independent reality exists yet concedes that we may, perhaps, fail in comprehending it, and when we fail, we just adopt the paradigm that is most *useful*.

## 1 WHAT DOES THIS ALL MEAN FOR MEDICAL EDUCATION RESEARCHERS?

Adopting a pragmatist stance can free the researcher from paradigmatic false dualist limitations. It can open up the researchers' inventory to exciting method‐mixing opportunities and provide better, more comprehensive answers in comparison to what quantitative or qualitative approaches can do in isolation.

When I was conducting research on self‐assessment of communication skills in clinical settings using a purist postpositivist deductive approach, I was able to examine score distributions and correlations between students' self‐assessment and external assessors' scores. This approach did not allow me, however, to examine the stories beneath the scores as in what the students felt and meant when self‐scoring and the contextual factors underlying agreement/disagreement with external scoring.

Adopting a pragmatist mixed‐methods approach allowed me to move beyond these limitations. It enabled me to address this *new* research question. Further, mixing an inductive qualitative approach (semi‐structured interviews) with the quantitative one permitted new factors affecting self‐assessment to come into focus whether they were student‐, interaction‐ or patient‐related factors. This pragmatist mixing generated findings with more explanatory power and actionable insights.

As illustrated, in pragmatism‐grounded research, you are encouraged to mix qualitative and quantitative methods when appropriate. Words can add meaning to numbers and numbers can add precision to words or identify patterns worth exploring in more (non‐numeric) depth; qualitative strengths offset quantitative weaknesses and vice versa; broader and more nuanced questions can be answered, and stronger conclusions can be drawn through convergence and corroboration of findings.^5^

We contend that methodological dualism in medical education may hinder the field's ability to fully capture the complexity of the socially embedded phenomena of medical education. Embracing pragmatism offers a path forward. A path that prioritises means most suited to answering research questions over dogmatic allegiances.

## AUTHOR CONTRIBUTIONS

**Ghaith Alfakhry**: Conceptualization; writing—original draft; writing—review and editing. **Danica Sims**: Supervision; writing—review and editing. **Ariel Lindorff**: Supervision; writing—review and editing.

## CONFLICT OF INTEREST STATEMENT

None of the authors have a conflict of interest to disclose.

## Data Availability

Data sharing is not applicable to this article as no datasets were generated or analysed during the current study.
